# Taylor on Genito-Urinary and Venereal Diseases and Syphilis

**Published:** 1900-12

**Authors:** 


TAYLOR ON GENITO-URINARY AND VENEREAL DISEASES AND SYPHILIS. The Pathology and Treatment of Genito-urinary and Venereal Diseases and Syphilis. By ROBERT W. TAYLOR, A. M., M. D., Clinical Professor of Venereal Diseases in the College of Physicians and Surgeons, New York. New (2d) edition. Octavo volume of 720 pages, with 135 engravings and 27 full-page plates in colors and monotone. Philadelphia and New York: Lea Brothers & Co. 1900. [Cloth, $5.00 net; leather, $6.00.]

The first edition of this work met with such general favor that a second volume became a necessity by reason of the advances made in genito-urinary work. This has given Dr. Taylor a chance to make a complete work on the one specialty in medicine which every man who has M. D. at the end of his name believes himself thoroughly familiar with, and about which so few have more than a mere smattering knowledge. Of course, Dr. Taylor advances his own views in connection with the treatment of acute diseases. He gives the procedures which have been most successful in his own practice; yet, for all that, he does not slight other well-recognised treatments, and while objecting to some he gives reasons and the reader is thus enabled to take his choice intelligently. Be they good or insufficient makes no difference; that is all a matter of personal experience anyway; the fact remains that the stamp of broad-minded honesty is given to Dr. Taylor's work by his treatment of these subjects, which is a decided relief when one considers how frequently authors are prone to give their own methods and condemn others, apparently on general principles.

A shining virtue of the book is that it is not over-burdened with morbidities, symptoms and conditions which are constant in well recognised abnormal states. These are not slighted, but one is not ding-donged in each succeeding chapter by a repetition of thinly disguised statements. Neither is there any disposition shown to parade rare and anomalous conditions and malformations found in unusual diseases. The work is in no sense a historical sketch and Dr. Taylor very wisely steers clear of dragging into a text-book any part of the genito-urinary curiosity shop. Out-of-date operations and treatments are ignored for the same reason. The work has been practically rewritten and much is added which is of decided benefit to it as a text-book. That portion dealing with genito-urinary operations is remarkable for its clearness, and a really excellent section is that devoted to the preparation of the patient and the instruments for operation, because it is practical, blunt, to the point and is utterly lacking in rhetorical prettiness: it is incisive. Dr. Taylor does not say, "it would be well to do this and that," he says, "do thus and so" and one feels that he means what he says and knows he is right when he says it.

The general arrangement, too, of the book is to be commended. There is nothing suggestive of indecision about it, no wading about, no splashing, no fancy frills, but it starts off and goes through the list of genito-urinary subjects in a straight line. Regarding gonorrhea, Dr. Taylor has fixed ideas and he puts them on paper with all the confidence of a man who believes he is right. Well, possibly he is, but he comes dangerously near the brink of fanaticism when he condemns, broadly and unreservedly, the irrigation method of Janet as a general proposition, under the sub-heading of "Fads in the Treatment of Gonorrhea." In a measure, he is right for he speaks harshly of the too heroic treatment of gonorrhea in the acute stage by these methods. It is easy to see that he is aiming at the general practising physician who, armed with a Valentine apparatus and a tin basin, sets himself up as a scientific foe to that unfortunate and painful reminder of a careless indiscretion. He calls it the "rapid transit" treatment, and calls attention to the fact that it is not generally a curative measure because with the cessation of the thick discharge the patient is permitted to depart "cured" while he is still afflicted with urinary evidences of urethral trouble. As a general proposition, this may be true enough when applied to the practising physician, but in the hands of one who has made gonorrhea a study, there is no doubt that the Janet method shortens the course of the disease infection, and then an experienced man orders the astringent treatment which Dr. Taylor outlines in his routine and carries the sorely distressed patient on to a complete and permanent recovery. The book is excellently illustrated and—charm of charms!—it is a volume in which there are no wood cuts of antiquated instruments. It is not a picture gallery of the Plebian ancestry of genito-urinary surgery. It is all up-to-date from cover to cover and one reason for its good mechanical construction is that Lea Brothers & Company are the publishers.—N. W. W.

